# The Assessment of Natural Cross Pollination Properties of a Novel Male-Sterile–Female-Fertile Mutation *ms_LC_01* in Soybean

**DOI:** 10.3390/plants12203538

**Published:** 2023-10-11

**Authors:** Wen Wang, Xiaojie Zhu, Yu Zhang, Huawei Gao, Zeru Zhang, Chunyan Yang, Yuhong Zheng, Qianqian Yu, Yixin Zhu, Yating Geng, Shulei Wang, Like Liu

**Affiliations:** 1 School of Life Sciences, Liaocheng University, Liaocheng 252059, China; 2 Shandong Xinfeng Seeds Co., Ltd., Liaocheng 252400, China; 3 Institute of Crop Sciences, Chinese Academy of Agricultural Sciences, Beijing 100081, China; 4 National Nanfan Research Institute (Sanya), Chinese Academy of Agricultural Sciences, Sanya 572024, China; 5 The Key Laboratory of Crop Genetics and Breeding of Hebei, Institute of Cereal and Oil Crops, Hebei Academy of Agriculture and Forestry Sciences, Shijiazhuang 050035, China; 6 Soybean Research Institute, Jilin Academy of Agricultural Sciences, Changchun 130033, China

**Keywords:** soybean, male sterility, *ms_LC_01*, natural outcrossing pollination

## Abstract

The value of a novel soybean male-sterile mutation *ms_LC_01* in breeding practice was determined by its outcrossing properties. Then, the effects of different planting arrangements on the pod set characteristics of male-sterile plants were assessed by using orthogonal experiments at two sites. At the same time, the effects of *ms_LC_01* male sterility on other traits were assessed in two C_2_F_2_ populations. In addition, the nectar secretion and natural outcross of male-sterile plants from four *ms_LC_01* lines were compared with one *ms1* line and one *ms6* line. The results of the orthogonal experiment showed that the pod numbers and pod set rates of male-sterile plants were decisively different between the two experimental sites but not between the two levels of the other factors. Both increasing the ratio of paternal parent to maternal parent and planting the parental seeds in a mixed way, the proportion of seeds pollinated by the target parent pollen could be increased. Except for the pod number per plant trait, there was no significant difference between male-sterile plants and their fertile siblings. The amount of nectar significantly differed among the lines. Compared with *ms1* and *ms6* male-sterile plants, the four *ms_LC_01* lines possessed significantly more or similar numbers of pod sets. The results of this study lay a foundation for the future use of this mutant in soybean breeding.

## 1. Introduction

Genic male-sterile–female-fertile (hereafter abbreviated as male-sterility) mutations are the basis of soybean recurrent selection, population improvement, and heterosis utilization [1,2,3,4,5]. For example, an elite variety has been bred using the *ms1* male-sterility mutation via recurrent selection [6]. To date, more than ten genic male-sterile mutants have been reported [7,8], with some, such as *ms1*, *ms2*, *ms3*, and *ms6*, broadly used in breeding practice [3,4].

Seed setting on the male-sterile plant is the key factor in determining the breeding value for a male-sterile mutation. Though it is usually a self-pollinated crop, soybean has typical nectary structures, which means it can be cross-pollinated by insects [9,10,11,12]. Therefore, any factor that can affect the nectar secretion or other characteristics attracting insects will affect the seed setting on the male-sterile plant. These factors come from genotype or the environment. For example, there is more than tenfold variation among the seed setting numbers for *ms2* loci with different genetic backgrounds [13]. The ways that nectar secretion and flower structure affect insect attraction vary among different soybean germplasms [14]. Furthermore, the insect attraction of soybean flowers may be related to secondary metabolites of volatile matter content [15]. As for environmental aspects, Robacker et al. [16] have shown that the air temperature, soil temperature, and soil nutrition can affect the size of the soybean flowers and the volume of nectar production, which both determine honey bee attraction. Recently, Qu et al. [17] demonstrated that rainfall, temperature, and light all influence the seed-setting rate of cytoplasmic male sterility (CMS). In addition to genotype and environmental factors, some planting arrangements can also affect the seed-setting rate of male-sterile plants, such as the ratio of pollen parents to pod parents and the removal of a fertile sibling in the line segregated for male-sterile plants [18].

Here, the male-sterile mutation locus designated *ms_LC_01* came from a male-sterile mutant called ms5053, which was induced via ethyl methanesulfonate (EMS) [19]. The male sterility of ms5053 is conferred by a single recessive gene, and it has shown good genetic stability over several years and across different locations; additionally, the *ms_LC_01* male-sterile plant has a good pod set property with different genetic backgrounds under natural field conditions [20]. In order to further evaluate the breeding value of *ms_LC_01* and improve its usability in breeding practice, an orthogonal experiment was carried out to study the effects of different field planting arrangements on the pod set characteristics of male-sterile plants under natural field conditions. At the same time, the pod set characteristics of *ms_LC_01* male-sterile plants were compared with those of the male-sterile plants of the *ms1* and *ms6* lines. Some nectar secretion characteristics of these male-sterile lines and the impact of *ms_LC_01* male sterility on other yield-related traits were additionally assessed.

## 2. Results

### 2.1. The Effects of Different Planting Arrangements on Pod Set Characteristics of ms_LC_01 Male-Sterile Plants in an Orthogonal Experiment

In the orthogonal experiment, the average pod number for the *ms_LC_01* male-sterile plants at the Shenxian site (36.084407 N, 115.594706 E) was 1.70 in 2020, with a range of 0~22. With different planting arrangements, the average pod number for the male-sterile plants ranged from 0.98 to 3.00 (Table 1). At the Longyao site (37.315049 N, 114.743717 E), the average pod number for the male-sterile plants was 7.50, with a range of 0~54. For different planting arrangements, it ranged from 5.50 to 11.68 (Table 1). Among different planting arrangements, the average pod set rate for male-sterile plants at the Longyao site was 11.61%, ranging from 7.29% to 18%, and at the Shenxian site it was 3.07%, with a range of 1.40%~4.92% (Table 1).

The results of the analysis of variance (ANOVA) showed that, except for the experimental site, the pod number and pod set rate for male-sterile plants were not significantly different between planting arrangement levels (Table 2 and Table 3, respectively).

### 2.2. The Effect of Planting Arrangements on the Seed Pollen Source of Male-Sterile Plants

In early December 2020, in order to advance the generation, seeds from the C_1_F_0_ generation harvested from the male-sterile plants were planted at Sanya, Hainan Province, China. In late February 2021, the C_1_F_1_ plants derived from the C_1_F_0_ seeds were threshed individually at Sanya. The numbers of harvested C_1_F_1_ plants from the SXT1, SXT2, SXT3, and SXT4 arrangements were all less than 10 and their data were not analyzed further because of their small sample sizes. Thanks to the fact that the hypocotyl color is linked to the flower color and the pollen donor, ‘Hedou12’ and ‘L85-1467’ both possessed a dominant purple flower, and the seed pollen source of the male-sterile plant was determined based on its hypocotyl color. At least four seeds per individual C_1_F_1_ plant were germinated in a Petri dish on wet filter paper. As only the seed that was pollinated by using the pollen from the target parent was usually needed (namely, ‘Hedou12’ or ‘L85-1467’), this kind of seed is hereafter called a target hybrid seed. The hypocotyl color observation results demonstrated that 60% of the seeds of male-sterile plants were target seeds (Table 4). The planting arrangement based on the ratio of paternal parents to maternal parents had the greatest influence on the target hybrid seed rate, followed by the method of planting and then the location and genotype of the paternal parent (Table 4).

Furthermore, 35 and 52 C_1_F_2_ lines derived from ‘Hedou12’ and ‘L85-1467’, respectively, were planted in the field in the summer of 2021 at the Shenxian site. During the growth period, we observed segregation in relation to the flower color and male sterility in all these lines, which proved the feasibility of using hypocotyl color to determine the pollen source of hybrid seeds.

The ANOVA results indicated that significant differences existed in the proportions of target hybrid seeds across planting arrangements (Table 5). The highest proportion of target hybrid seeds was in SXT6 (79.66%) at the Shenxian site and the lowest was in LYT1 (34.97%) at the Longyao site. The proportions of the LYT1 and LYT2 treatments were significantly lower than those of the SXT6 and LYT3 treatments at *p* = 0.01 (Table 4).

A *t*-test was used to assess the effects of planting arrangements on the pollen sources of the hybrid seeds of male-sterile plants: 71.23% of hybrid seeds of the male-sterile plants were target seeds in the arrangement with a 5:1 ratio of paternal parents to maternal parents, which was conclusively higher than the value of 36.27% at the 1:1 level (*p* < 0.0001). Similarly, the proportion of target hybrid seeds in the arrangement with mixed planting of paternal and maternal parents was higher than that in the arrangement where the paternal and maternal parents were planted separately (*p* = 0.047) (Table 4). However, the proportions of target hybrid seeds did not differ between the two levels of the other factors.

### 2.3. The Influence of ms_LC_01 Male Sterility on other Agronomic Traits

**Figure 1:**
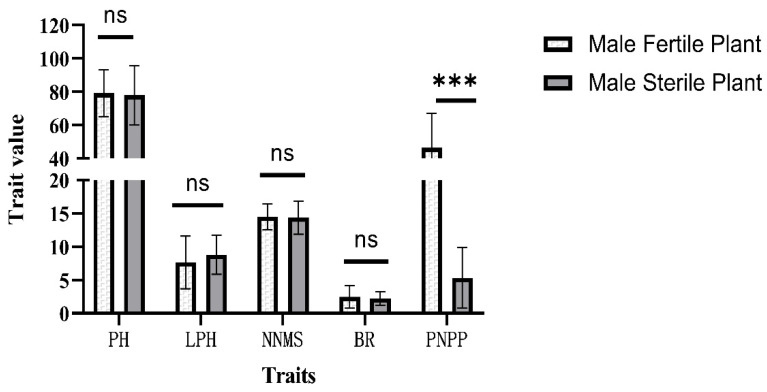
*t*-test of several traits with male-sterile plants and male-fertile plants in the HC_2_F_2_ population, segregating the population for *ms_LC_01*. Error bar, one standard deviation. ***, *p* < 0.001. ns, not significant. PH, plant height. LPH, lowest pod height. NNMS, node number on the main stem. BR, branch number. PNPP, pod number per plant.

**Figure 2:**
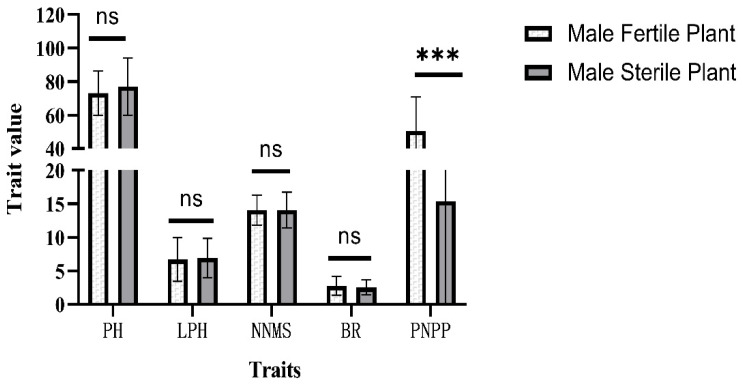
*t*-test of several traits with male-sterile plants and male-fertile plants in the LC_2_F_2_ populations, segregating the population for *ms_LC_01*. Error bar, one standard deviation. ***, *p* < 0.001. ns, not significant. PH, plant height. LPH, lowest pod height. NNMS, node number on the main stem. BR, branch number. PNPP, pod number per plant.

In autumn 2021, the seeds of male-sterile plants, which, in the C_1_F_2_ generation, were derived from ‘L85-1467’ and ‘Hedou12’, were harvested and propagated at Sanya. In the summer of 2022, individual plants from the C_2_F_2_ generation were selected for further planting in a row if they possessed more than 40 seeds. In the autumn, 30 and 37 rows segregating for male-sterile plants were harvested from the HC_2_F_2_ and LC_2_F_2_ populations, respectively. On average, 5.23 and 5.03 male-sterile plants per row from the HC_2_F_2_ and LC_2_F_2_ populations, respectively, and more than 5 normal fertile plants per row were tested for some yield-related traits, including pod numbers per plant, plant height, lowest pod height, node numbers on the main stem, and branch numbers. The *t*-test results showed that, except for the pod number per plant trait, the other traits were not significantly different between male-sterile plants and normal male-fertile plants, indicating that the *ms_LC_01* loci only affected the plant pod number (Figure 1 and Figure 2).

### 2.4. Comparison of Pod Set Characteristics among the ms_LC_01, ms1, and ms6 Male-Sterile Lines with Different Genetic Backgrounds under Natural Field Conditions

**Figure 3:**
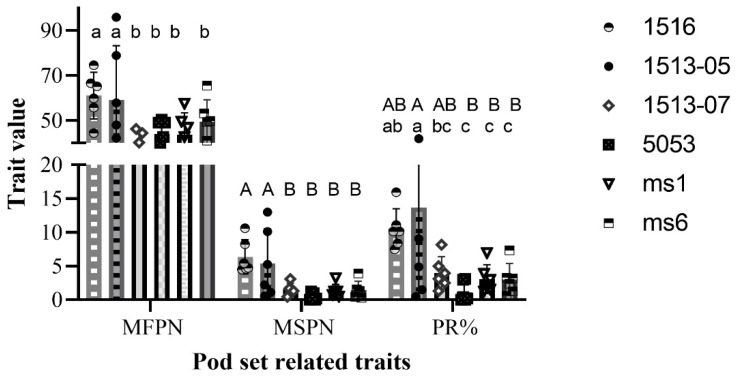
Significance analysis of pod number-related traits among *ms_LC_01*, *ms1*, and *ms6* male-sterile lines. Error bar, one standard deviation. MFPN, male-fertile plant pod number. MSPN, male-sterile plant pod number. MSPR, male-sterile plant pod set rate. The same uppercase letter means no significant difference at *p* < 0.01, and the same lowercase letter means no significant difference at *p* < 0.05.

**Figure 4:**
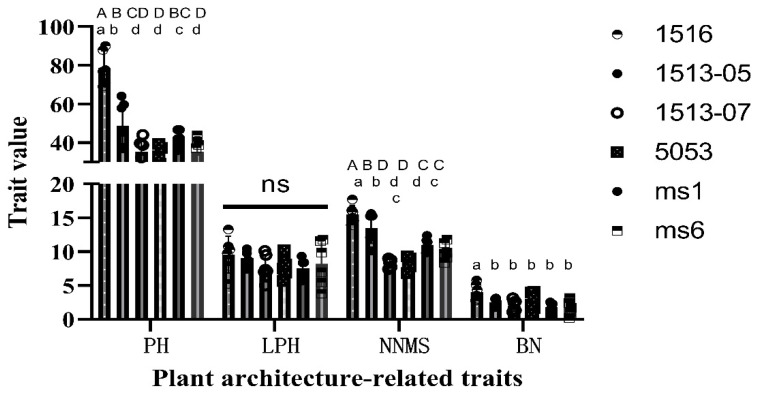
Significance analysis of plant type-related traits among *ms_LC_01*, *ms1*, and *ms6* male-sterile lines. Error bar, one standard deviation. PH, plant height. LPH, lowermost pod height. NNMS, node number on the main stem. BN, branch number. The same uppercase letter means no significant difference at *p* < 0.01, and the same lowercase letter means no significant difference at *p* < 0.05.

In order to assess the value of *ms_LC_01* male sterility in breeding practice, the pod set characteristics of male-sterile plants of four *ms_LC_01* lines (1513-05, 1513-07, 1516, and 5053) were compared with those of one *ms1* line and one *ms6* line. These lines were planted by using a randomized complete block design with three replicates at the east campus of Liaocheng University and the Shenxian site, respectively, in 2022. On average, 10.33 male-sterile plants and 6.04 male-fertile plants per genotype per repeat were tested for pod number-related traits and plant type-related traits, respectively. As the original pod number and pod set rate data for the male-sterile plants did not meet the requirements of the ANOVA for the normal distribution or homogeneity of variance across different factor levels (Shapiro–Wilk test *p* < 0.001, Bartlett test *p* < 0.001), log10(x + 1) transformation for the pod number and arcsine transformation for the pod setting rate of the male-sterile plants were carried out, and then the transformed data were used for the ANOVA (Table 6 and Table 7). The ANOVA results showed that the differences in the pod numbers and pod set rates of the male-sterile plants were significant among genotypes and sites but not among replicates. Furthermore, the interactions between genotypes and sites or replicates had no effects on these two traits (Table 6 and Table 7). The results of Duncan’s multiple tests showed that the pod number of male-sterile line 1516 (6.35 pods/plant) was significantly higher than those of other male-sterile lines at both the *p* = 0.05 and *p* = 0.01 levels. Moreover, the highest pod set rate (13.64%) was observed in line 1513-05, which was also significantly higher than other male-sterile lines. The pod number and pod set rate of male-sterile plants of line 5053 were both significantly lower than those of the other three genotypes at both the *p* = 0.05 and *p* = 0.01 level, indicating that genetic background had a great influence on the natural outcrossing characteristics of male-sterile plants in the field (Figure 3 and Figure 4).

### 2.5. Comparison of Nectar Secretion Characteristics among ms_LC_01, ms1, and ms6 Male-Sterile Lines

**Figure 5:**
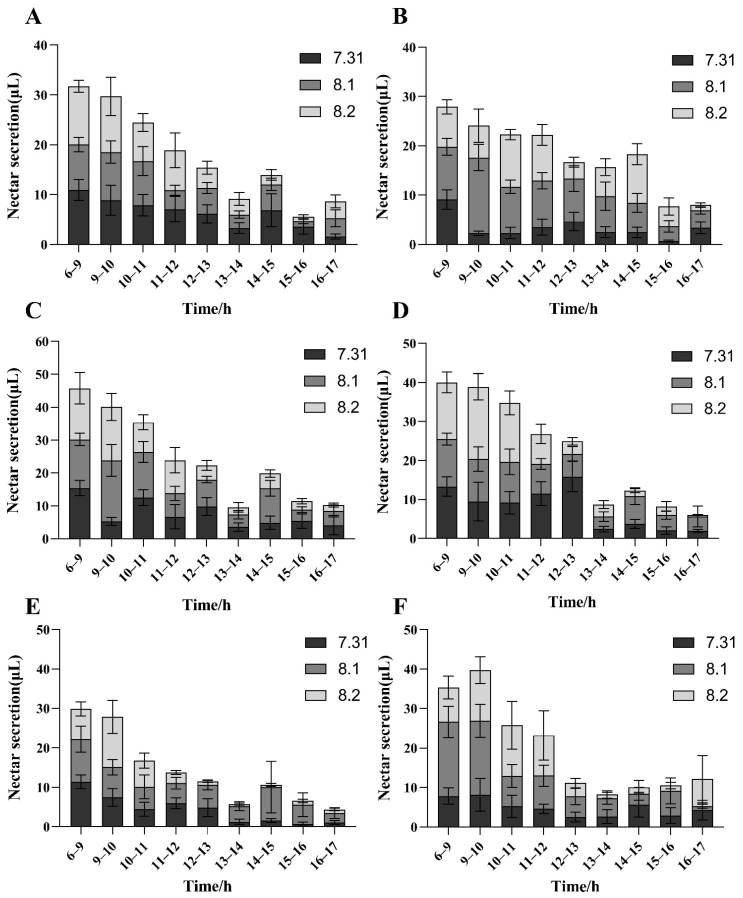
Daily variations in nectar secretion of *ms_LC_01*, *ms1*, and *ms6* male-sterile lines for three consecutive days. Error bar, one standard deviation. (**A**) 1516 line, (**B**) 5053 line, (**C**) 1513-05 line, (**D**) 1513-07 line, (**E**) *ms1* line, (**F**) *ms6* line.

In order to explore the cause underlying the variation in pod set characteristics among male-sterile lines, the nectar secretion of these lines was measured. In every time period, the nectar secretion of two flowers at a time was determined with ten repeats for each genotype. The results of the nectar secretion measurements indicated that four *ms_LC_01* male-sterile lines and one *ms1* male-sterile line showed that the daily nectar secretion rules were highly consistent. Specifically, a nectar secretion peak occurred from 6:00 to 9:00 in the morning followed by a gradual decrease and a small sub-peak in nectar secretion from 14:00 to 15:00 in the afternoon (Figure 5). On the other hand, the nectar secretion peak of the *ms6* male-sterile line occurred from 9:00 to 10:00 in the morning, followed by a gradual decline until it stabilized from 14:00 to 15:00 in the afternoon. Broadly speaking, the nectar secretion peak appeared from 6:00 a.m. to 10:00 a.m.

**Figure 6:**
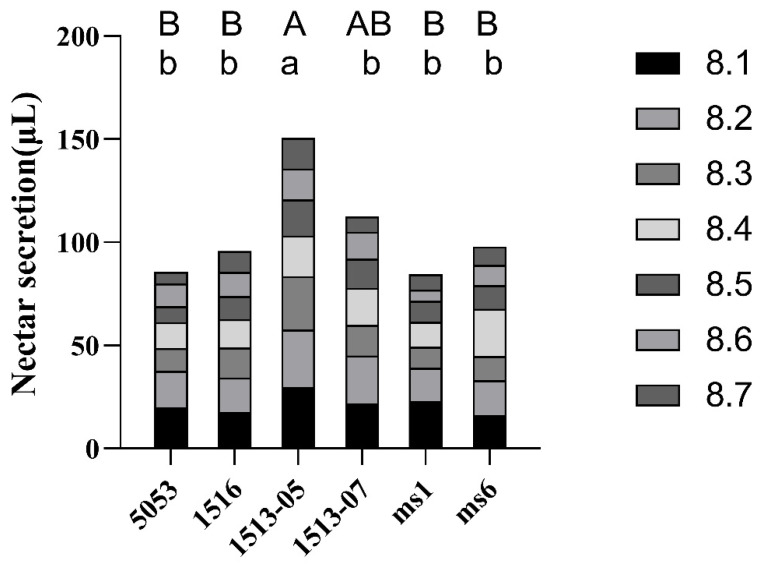
Nectar secretion of *ms_LC_01*, *ms1*, and *ms6* male-sterile lines for seven consecutive days. The same uppercase letter means no significant difference at *p* < 0.01, and the same lowercase letter means no significant difference at *p* < 0.05.

Subsequently, the nectar quantities of these male-sterile lines were measured for seven consecutive days at the peak time. There were significant differences in nectar secretion among the different lines. The 1513-05 line possessed significantly higher nectar secretion compared to the other lines. The nectar quantities of the four *ms_LC_01* lines were either significantly higher than or similar to those of the *ms1* and *ms6* male-sterile lines (Figure 6). Combined with the pod set data for the male-sterile plants presented above, a significant correlation was found between pod set rate and nectar secretion amount (r = 0.91, *p* = 0.012) but not between pod number and nectar secretion amount (r = 0.73, *p* = 0.099).

### 2.6. Correlations between Pod Set Characteristics and Other Traits in Different Populations

In the male-sterile lines, a conclusively positive correlation existed between the pod numbers of the male-sterile plants and plant height (r = 0.81, *p* < 0.001), as well as the number of main stem nodes (r = 0.84, *p* < 0.001). The pod numbers of the male-sterile plants were also significantly correlated with the branch numbers (r = 0.34, *p* = 0.04), but not the bottom pod height or the pod numbers of the fertile plants.

In the HC_2_F_2_ population, there was a significant positive correlation between the pod numbers of male-sterile plants and plant height (r = 0.39, *p* = 0.03), as well as branch numbers (r = 0.37, *p* = 0.04). Meanwhile, a decisively negative correlation existed between the pod numbers of male-sterile plants and those of fertile plants (r = −0.50, *p* = 0.005). In the LC_2_F_2_ population, a decisively positive correlation (r = 0.45, *p* = 0.005) existed between the pod numbers of male-sterile plants and the numbers of main stem nodes (Table 8). Although the correlation between pod number and other traits was not completely consistent in these populations, plant luxuriance was generally positively correlated with the pod numbers of male-sterile plants, indicating that a tall and luxuriant male-sterile plant usually produced more pods. For male-sterile plants, regardless of the population, the pod set rate had a correlation coefficient greater than 0.70 regarding the pod number, suggesting that the primary determinant of the pod set rate was the pod number.

## 3. Discussion

### 3.1. Effects of Planting Arrangements on Pod Set Characteristics of Male-Sterile Plants under Natural Field Conditions

Wang et al. [21] and Zhao et al. [22] have demonstrated that some planting arrangement variables, such as the ratio of paternal rows to maternal rows and parent arrangement within in-line rows, and planting methods can affect the pod set rate of soybean cytoplasmic male-sterile lines. In this study, the natural pod numbers and pod set rates of *ms_LC_01* male-sterile plants did not vary significantly between the two levels of the different arrangements. The reason may be attributed to our exclusive focus on the pod set numbers of male-sterile plants without taking into account the pollen source. Alternatively, it could be related to variations in materials and ecological environments. In 2020, the pod numbers per male-sterile plant were significantly higher at the Longyao experimental site than at the Shenxian experimental site, indicating that environmental factors have a significant impact on the natural outcrossing characteristics of male-sterile plants. This finding is consistent with the results for cytoplasmic male-sterile lines of sesame [23] and soybean [24]. Qu et al. [17] also demonstrated the impact of rainfall and light intensity on the outcrossing rate and hybrid seed yield of soybean cytoplasmic male-sterile lines. In this paper, the pod numbers and pod set rates of male-sterile plants both positively correlated with the field growth of soybeans, suggesting that the environment may play a role in the pod set characteristics of male-sterile plants by influencing soybean growth.

### 3.2. Effects of Different Field Planting Arrangements on the Proportion of Target Hybrid Seeds in the Male-Sterile Plants

It has been shown that the number of target hybrid seeds in male-sterile plants linearly increases as the pollen donors increase in intermating blocks [25,26]. In this study, a similar result was found; namely, when the ratio of paternal parents to maternal parents increased from 1:1 to 5:1, the target hybrid seed proportion increased from 36.27% to 71.63%, indicating that increasing the paternal parent proportion could enhance the opportunity to pollinate the flower in male-sterile plants with the pollen from the paternal parents. On the other hand, when the seeds of the paternal parent and maternal parent were mixed and planted in the same row rather than in different rows separately, the target hybrid seed proportion was 70.27% instead of 53.95%, which is consistent with the result from Zhao’s study [22], where planting the paternal parent and cytoplasmic male-sterile line in the same hole markedly increased the pod numbers per cytoplasmic male-sterile plant compared with planting the parental lines in rows separately.

St. Martin and Ehounou [27] reported that significant variations existed among varieties in terms of pollen contribution to the seed set of male-sterile plants. Yan et al. [28] also found the seed set rate of the cytoplasmic male-sterile line varied significantly with different maintainer lines, indicating that different paternal parents have different levels of attractiveness for insects. However, in the present study, the pollen donors ‘L85-1467’ and ‘Hedou12’ both supplied similar amounts of pollen for the seeds of male-sterile plants, which may have been due to the similar attractiveness to insects of these two pollen donors.

### 3.3. Effects of the ms_LC_01 Male-Sterility Mutation on Other Traits

A lack of adverse genetic effects or of a link with an adverse allele is the prerequisite in breeding practice for a male-sterile allele. However, in practical scenarios, certain male-sterile mutant alleles exhibit adverse genetic effects. For instance, various studies have demonstrated that the *ms1* male-sterile mutant allele can induce polyploidy [29,30,31], that the expression of *msp* male sterility is influenced by environmental factors [32], that the photoperiod regulates the mutation of *ms3* male sterility [33], and that temperature affects the expression of *ms4* male sterility [31]. As for the male-sterile mutation allele *ms_LC_01* in this study, Zhang et al. [20] demonstrated that this male-sterility mutation can be inherited steadily across different years and locations. In this study, there were no significant differences in yield-related traits between male-sterile plants and male-fertile plants within the two C_2_F_2_ populations when segregating for male-sterile plants, primarily indicating that *ms_LC_01* male-sterile mutation had no obvious adverse genetic effects and that it is a promising mutation for breeding practice in the future.

### 3.4. Comparison of Pod Set Characteristics of ms_LC_01 Lines with those of ms1 and ms6 Lines

The pod set characteristics of male-sterile lines are another crucial factor for their availability in breeding practice. In this study, the pod numbers and pod set rates of the male-sterile plants of four *ms_LC_01* lines were both significantly higher than or similar to those of *ms1* and *ms6* lines under completely natural field conditions. Additionally, in the HC_2_F_2_ and LC_2_F_2_ populations, the average pod numbers for each male-sterile plant were 5.33 and 7.04, respectively, which were also significantly higher than those of *ms1* and *ms6* male-sterile plants (*p* < 0.001). In a study involving the release of alfalfa leafcutter bees in the field, Ortiz-Perez et al. [13] found that *ms1* male-sterile plants (North Carolina) produced 2.17 seeds per plant, while *ms6* male-sterile plants (Ames-2) yielded 4.12 seeds per plant. Graybosch et al. [31], on the other hand, reported that the average male-sterile *ms1* plant (Clark) yielded 3.9 seeds per plant. In this study, relying solely on natural insect pollinators, each *ms1* male-sterile plant produced an average of 1.29 pods with approximately 2.6 grains per plant (calculated from an average of 2 pods per plant) [10,34]. The pod numbers of both *ms1* and *ms6* male-sterile plants in this study were comparable to previous studies. Thus, it can be stated that, in this study, the pod set numbers of *ms_LC_01* male-sterile plants were higher than or similar to those of *ms1* and *ms6* male-sterile plants, further indicating the good application potential of *ms_LC_01* male-sterile mutation in breeding practice.

### 3.5. Nectar Secretion Characteristics of Different Male-Sterile Lines

The nectar secretion peak for the six male-sterile lines mainly occurred between 6:00 and 9:00 in the morning, which was consistent with previous studies [15]. There were significant differences in nectar secretion among the different lines, and other studies also demonstrated that the genotype background can considerably affect the nectar secretion [15,35,36]. In this study, the pod set rate of male-sterile plants was significantly positively correlated with the nectar secretion amount, indicating that nectar secretion is an important attractor for insects, similarly to the results from other reports [15,37].

## 4. Materials and Methods

### 4.1. Experimental Material

#### 4.1.1. *ms_LC_01* Male-Sterile Lines Segregating for Male-Sterile Plants

There were four *ms_LC_01* male-sterile lines segregated for male-sterile plants; namely, 5053, 1516, 1513-05, and 1513-07. Among them, the 5053 line was a compound mutant derived from the ‘Jidou17’ genetic background, which exhibited dwarfism and male sterility. The other three lines were all derived from natural hybrid seeds of the 5053 male-sterile plants and selected for more than six generations. The 5053 and 1513-05 lines were dwarf male-sterile lines with a plant height of approximately 30 cm, while the 1513-07 line was a semi-dwarf male-sterile line with a plant height of around 50 cm. The 1516 line was a comparably tall male-sterile line, reaching about 80 cm in height. These four male-sterile lines all had white flowers.

#### 4.1.2. Pollen Donor

Two germplasms, ‘L85-1467’ and ‘Hedou12’, both with purple flowers, were provided by the Institute of Crop Science, Chinese Academy of Agricultural Sciences. They had similar growth stages as the *ms_LC_01* male-sterile line and were used as pollen donors in the orthogonal experiment.

#### 4.1.3. *ms1* and *ms6* Male-Sterile Lines Segregating for Male-Sterile Plants

The *ms1* and *ms6* male-sterile soybean lines segregated for male-sterile plants were provided by the Key Laboratory of Crop Genetics and Breeding of Hebei, Institute of Cereal and Oil Crops, Hebei Academy of Agriculture and Forestry Sciences, Shijiazhuang, China, and the Soybean Research Institute, Jilin Academy of Agricultural Sciences, Changchun, China, respectively.

### 4.2. Methods

#### 4.2.1. The Effects of Planting Arrangements on Pod Set Characteristics of *ms_LC_01* Male-Sterile Plants

In this experiment, we adopted an orthogonal experimental design without considering the interaction between factors. In late June 2020, eight arrangements were prepared in Shenxian County (36.084447 N, 115.594706 E), Shandong Province, China (hereinafter referred to as the Shenxian site), and four arrangements were prepared in Longyao County (37.315049 N, 114.743717 E), Hebei Province, China (hereinafter referred to as the Longyao site) (Table 9 and Table 10). For field planting, the lines were set at a length of 5 m with rows spaced 50 cm apart and plant spacing set at 7 cm. Additionally, 15 m of corn was planted between the two arrangements for isolation. Due to an insufficient quantity of seeds from any single line, equal numbers of seeds from four lines (namely, 5053, 1516, 1513-05, and 1513-07) were mixed to supply male-sterile plants.

Considering previous studies [18,21,22] and the convenience of field planting, the field planting arrangements primarily involved four factors; namely, the ratio of paternal lines to maternal lines, the paternal genotype, the planting method for the parental line, and whether the fertile plant was removed from the male-sterile lines segregating for male-sterile plants or not. Specifically, an L_8_(2^7^) orthogonal table was used in the field experiment at the Shenxian site with four factors at two levels (Table 9).

These four factors are described in detail as follows:

The ratios of the paternal lines to the maternal lines were 1:1 (level one) and 5:1 (level two) based on seed number;

The paternal genotypes were Hedou12 (level one) and L85-1467 (level two);

The planting method for the parental line involved two levels. Level one was planting the paternal lines and maternal lines separately in rows; level two was planting the parental lines in the same row;

Removing the fertile plant from the male-sterile lines segregating for male-sterile plants was set as level one and no removal was set as level two. Regrettably, the implementation of this process was impeded due to the COVID-19 pandemic.

The field experiment conducted at the Longyao experimental site involved three factors with two levels each and was designed using the L_4_(2^3^) orthogonal table without considering factor interactions. The three factors were the same as the first three factors in the orthogonal design at the above Shenxian site (Table 10).

At the R8 stage of soybean development [38], in accordance with [39,40], the plants with green leaves, many fleshy pods without seeds on the stem, and without or with few normal pods, which may come from outcrossing on the stem, were regarded as the male-sterile plants. The normal plants possessed a normal number of mature pods and were deciduous.

#### 4.2.2. Determination of the Pollen Sources for Seeds on the Male-Sterile Plants in the Orthogonal Experiment

In this experiment, the pollen sources for the seeds of male-sterile plants were either the target paternal parent (‘Hedou12’ or ‘L85-1467’) or the fertile sibling plant of the male-sterile lines segregating for male-sterile plants. As only those seeds pollinated by the target parent are considered as target hybrid seeds in breeding practice, the pollen source for the hybrid seeds in male-sterile plants was determined in terms of whether there was segregation for hypocotyl color, flower color, or male sterility among the descendants. As the natural outcrossing process in male-sterile plants in this experiment was similar to a recurrent selection process, the letter “C” was used to indicate outcross times to distinguish materials from different generations. In the beginning of December 2020, the seeds of male-sterile plants from the C_1_F_0_ generation were propagated at Sanya, Hainan Province, China. The C_1_F_1_ plants were threshed individually at the R8 developmental stage in March 2021. Four seeds in the C_1_F_2_ generation, which derived from a single C_1_F_1_ plant, were selected for a germination test to observe hypocotyl color. As the hypocotyl color is linked to flower color and the target paternal parents (‘Hedou12’ and ‘L85-1467’, both with purple flowers), the presence of one or more seedlings with a purple hypocotyl color in the germination test indicated that the pollen for a hybrid seed from a male-sterile plant was from the target paternal parent. To mitigate the occurrence of low-probability events, two additional seeds were further employed for germination if all four seedlings possessed green hypocotyls. Meanwhile, to prevent the admixture of other purple flower germplasms in the individual harvested C_1_F_1_ plants, at least 20 seeds with the C_1_F_2_-generation genotype were sown into rows in late June 2021. If a C_1_F_1_ plant came from a target hybrid seed of a male-sterile plant, segregation for hypocotyl color, flower color, or male-sterile plants occurred; otherwise, it was considered as an admixture.

#### 4.2.3. The Relationship between *ms_LC_01* Male-Sterility Mutation and other Traits

In autumn 2021, male-sterile plant seeds were harvested from the C_1_F_2_ population derived from the target hybrid seeds with ‘Hedou12’ or ‘L85-1467’ as pollen donors. These seeds were then propagated at Sanya, Hainan Province, China, and C_2_F_1_ plants were later threshed individually at the R8 stage. In the summer of 2022, the C_2_F_2_ populations segregating for male-sterile plants were obtained by sowing C_2_F_1_ plant seeds at the Shenxian site. The progeny of each pollen donor were planted with 50 rows of C_2_F_1_ individual plants 2 m in length, with 50 cm row spacing and 5 cm plant spacing. In autumn 2022, all male-sterile plants and more than five normal fertile plants in rows with more than twenty plants were examined for plant height, low pod height, number of main stem nodes, number of effective branches, and pod numbers per plant.

#### 4.2.4. Comparison of Pod Set Characteristics of *ms_LC_01* Male-Sterile Plants with those of *ms1* and *ms6* Male-Sterile Plants

The male-sterile lines, including four *ms_LC_01* lines, one *ms1* line, and one *ms6* line, were planted in the experimental field of the College of Life Sciences at Liaocheng University on 24 June 2022 and at the Shenxian site on 7 July 2022. Each male-sterile line was arranged in five rows with a length of 2 m, row spacing of 50 cm, and plant spacing of 5 cm. The arrangement was randomized into complete blocks with three replicates. Pod set characteristics were investigated for each male-sterile plant as described in Section 4.2.1.

#### 4.2.5. Investigation of the Nectar Secretion Characteristics in the Male-Sterile Lines *ms_LC_01*, *ms1*, and *ms6*

In the experimental field of the College of Life Sciences at Liaocheng University, we measured daily variations in the nectar secretion of four *ms_LC_01* lines, one *ms1* line, and one *ms6* line for three consecutive days at the R2 stage according to the method described by Zhang et al. [15]. Subsequently, the nectar secretions were measured for seven consecutive days only at the peak time.

### 4.3. Data Analysis

The raw data were primarily processed using EXCEL2010. Statistics analysis was carried out with SPSS 17.0. When performing ANOVA analysis, the Shapiro–Wilk method was employed to test for conformity with the normal distribution assumptions, and the Bartlett method was used to assess the homogeneity of variance. Data that did not meet the requirements for ANOVA analysis were transformed using either log(10) (x+1) or arcsine, where x was the original datum.

## 5. Conclusions

Without eliminating the fertile sibling plants, *ms_LC_01* male-sterile plants bore similar numbers of pods with the two levels of the different planting arrangement factors, except for the planting locations. Broadly speaking, the pod numbers of male-sterile plants are positively related to plant growth; specifically, better growth conditions for male-sterile plants mean more pods. Increasing the ratio of paternal parents to maternal parents and planting the parents in a mixed way can both increase the target hybrid seed rates of male-sterile plants. Compared with *ms1* and *ms6* male-sterile plants, *ms_LC_01* male-sterile plants bore more or similar numbers of pods.

## Figures and Tables

**Table 1:** Pod number per male-sterile plant and pod set rate for *ms_LC_01* sterile plants at the Shenxian and Longyao sites.

Location	Arrangement	Number of Male-Sterile Plants	Pod Number for Male-Sterile Plants	Range of Pod Numbers for Male-Sterile Plants	Pod Set Rate for Male-Sterile Plants (%)
Shenxian site	SXT1	39	1.82	0~9	3.01
	SXT2	33	1.00	0~7	1.75
	SXT3	45	0.98	0~8	1.40
	SXT4	37	1.22	0~6	2.33
	SXT5	45	1.78	0~11	3.40
	SXT6	34	3.00	0~22	4.92
	SXT7	33	1.88	0~6	4.39
	SXT8	27	1.93	0~19	3.38
	Average	36.63	1.70	0~11	3.07
Longyao site	LYT1	31	11.68	0~54	18.00
	LYT2	30	5.50	0~19	9.62
	LYT3	39	6.08	0~35	7.29
	LYT4	27	6.74	0~32	11.52
	Average	31.75	7.50	0~35	11.61

**Table 2:** ANOVA of the pod number for *ms_LC_01* male-sterile plants.

Source	Degrees of Freedom	Sum of Squares	Mean Square	F Value	*p* Value
Location	1	89.68	89.68	27.61	0.0019
Genotype of paternal parent	1	2.92	2.92	0.90	0.38
Ratio of paternal parent to maternal parent	1	0.89	0.89	0.27	0.62
Method of planting	1	1.94	1.94	0.60	0.47
Error	6	19.49	3.25		
Total	11	116.77			

**Table 3:** ANOVA of the pod set rate for *ms_LC_01* male-sterile plants.

Source	Degrees of Freedom	Sum of Squares	Mean Square	F Value	*p* Value
Location	1	187.49	187.49	21.95	0.002
Genotype of paternal parent	1	7.19	7.19	0.84	0.39
Ratio of paternal parent to maternal parent	1	1.48	1.48	0.17	0.69
Method of planting	1	0.83	0.83	0.10	0.76
Error	7	59.80	8.54		
Total	11	256.80			

**Table 4:** Range analysis of the effects of planting arrangements on target hybrid seed rate of male-sterile plants in the orthogonal experiment.

Arrangement	Location	Ratio of Paternal Parent to Maternal Parent	Genotype of Paternal Parent	Method of Planting	Target Hybrid Seed Rate (%)
LYT1	1	1	1	1	34.96 cB
LYT2	1	1	2	2	38.85 bcB
LYT3	1	2	1	2	79.09 aA
LYT4	1	2	2	1	70.79 aAB
SXT5	2	2	1	1	55.11 abcAB
SXT6	2	2	1	2	79.67 aA
SXT7	2	2	2	1	65.48 aAB
SXT8	2	2	2	2	62.22 abAB
K1	60.16	36.27 A	64.10	53.95 a	
K2	67.17	71.63 B	60.60	70.27 b	
R	7.01	35.36	3.49	16.32	

Note: The same lowercase letter means no significant difference at *p* = 0.05 and the same uppercase letter means no significant difference at *p* = 0.01.

**Table 5:** ANOVA for the rate of target pollination on male-sterile plants among different arrangements.

Source	Degrees of Freedom	Sum of Squares	Mean Square	F Value	*p* Value
Arrangements	7	0.69	0.099	6.19	0.01
Error	16	0.26	0.016		
Total	23	0.95			

**Table 6:** ANOVA for pod number per male-sterile plant from the *ms_LC_01*, *ms1*, and *ms6* lines.

Source	Degrees of Freedom	Sum of Squares	Mean Square	F Value	*p* Value
Genotype	5	2.03	0.41	14.60	<0.0001
Location	1	0.50	0.50	17.90	0.001
Repeat	2	0.01	0.004	0.16	0.85
Genotype × location	5	0.32	0.06	2.31	0.11
Genotype × repeat	10	0.26	0.03	0.94	0.53
Error	12	0.33	0.03		
Total	35	3.45			

**Table 7:** ANOVA for the pod set rates of the male-sterile plants from the *ms_LC_01*, *ms1*, and *ms6* lines.

Source	Degrees of Freedom	Sum of Squares	Mean Square	F Value	*p* Value
Genotype	5	0.29	0.06	7.03	0.003
Location	1	0.11	0.11	12.76	0.004
Repeat	2	0.002	0.001	0.10	0.902
Genotype × location	5	0.12	0.02	2.96	0.057
Genotype × repeat	10	0.07	0.01	0.79	0.640
Error	12	0.10	0.01		
Total	35	0.69			

**Table 8:** The coefficients between pod numbers per male-sterile plant and other traits.

Population	Trait	Plant Height	Lowest Pod Height	Node Numbers on Main Stem	Branch Number	Pod Numbers per Male-Fertile Plant	Pod Set Rate of Male-Sterile Plant
*ms_LC_01*, *ms1*, and *ms6* lines	Pod numbers per male fertile plant	0.81 ***	0.28 ns	0.84 ***	0.34 *	0.10 ns	0.95 ***
	Pod set rate of male-sterile plants	0.69 ***	0.29 ns	0.72 ***	0.23 ns	−0.11 ns	1
HC_2_F_2_	Pod numbers per male fertile plant	0.39 *	−0.09	0.26	0.37 *	−0.50 **	0.73 **
	Pod set rate of male-sterile plants	0.18	−0.13	0.16	0.50 **	−0.64 **	1
LC_2_F_2_	Pod numbers per male fertile plant	0.19	−0.26	0.45 **	0.08	0.14	0.82 **
	Pod set rate of male-sterile plants	0.06	−0.17	0.28	−0.03	−0.19	1

Note: * *p* < 0.05; ** *p* < 0.01; *** *p* < 0.0001.

**Table 9:** Orthogonal experiment design for pod set characteristics of *ms_LC_01* male-sterile plants at the Shenxian site.

Arrangement	Ratio of Paternal Parents to Maternal Parents	Genotype of Paternal Parent	Planting Method for the Parental Lines	Removal of Fertile Sibling
SXT1	1	1	1	1
SXT 2	1	1	2	2
SXT 3	1	2	1	1
SXT4	1	2	2	2
SXT 5	2	1	1	1
SXT 6	2	1	2	2
SXT 7	2	2	1	1
SXT 8	2	2	2	2

**Table 10:** Orthogonal experiment design for pod set characteristics of *ms_LC_01* male-sterile plants at Longyao site.

Arrangement	Ratio of Paternal Parents to Maternal Parents	Genotype of the Paternal Parent	Method of Planting
LYT1	1	1	1
LYT2	1	2	2
LYT 3	2	1	2
LYT 4	2	2	1

## Data Availability

All the data included in the main text.
